# Methanol and Ethanol Electrooxidation on ZrO_2_/NiO/rGO

**DOI:** 10.3390/nano13040679

**Published:** 2023-02-09

**Authors:** Mohammad Bagher Askari, Hadi Beitollahi, Antonio Di Bartolomeo

**Affiliations:** 1 Department of Semiconductor, Institute of Science and High Technology and Environmental Sciences, Graduate University of Advanced Technology, Kerman 7631818356, Iran; 2 Environment Department, Institute of Science and High Technology and Environmental Sciences, Graduate University of Advanced Technology, Kerman 7631885356, Iran; 3 Department of Physics “E. R. Caianiello” and “Interdepartmental Center NANOMATES”, University of Salerno, Fisciano 84084, Salerno, Italy

**Keywords:** metal oxides, reduced graphene oxide, ZrO_2_/NiO/rGO, methanol oxidation reaction, ethanol oxidation reaction

## Abstract

Recently, transition metal oxides have been considered for various applications due to their unique properties. We present the synthesis of a three-component catalyst consisting of zirconium oxide (ZrO_2_), nickel oxide (NiO), and reduced graphene oxide (rGO) in the form of ZrO_2_/NiO/rGO by a simple one-step hydrothermal method. X-ray powder diffraction (XRD), scanning electron microscope (SEM), and bright-field transmission electron microscopy (BF-TEM) analyses were performed to accurately characterize the catalysts. Cyclic voltammetry (CV), electrochemical impedance spectroscopy (EIS), and linear sweep voltammetry (LSV) analyses were also carried out to investigate the methanol and ethanol alcohol electrooxidation ability of the synthesized nanocatalysts. Inspired by the good potential of metal oxides in the field of catalysts, especially in fuel-cell anodes, we investigated the capability of this catalyst in the methanol oxidation reaction (MOR) and ethanol oxidation reaction (EOR). After proving the successful synthesis and examining the surface morphology of these materials, detailed electrochemical tests were performed to show the outstanding capability of this new nanocatalyst for use in the anode of alcohol fuel cells. ZrO_2_/NiO/rGO indicated a current density of 26.6 mA/cm^2^ at a peak potential of 0.52 V and 99.5% cyclic stability in the MOR and a current density of 17.3 mA/cm^2^ at a peak potential of 0.52 V and 98.5% cyclic stability in the EOR (at optimal concentration/scan rate 20 mV/s), representing an attractive option for use in the anode of alcoholic fuel cells.

## 1. Introduction

Modern society and industry are highly dependent on electricity produced by fossil fuels for consumer and manufacturing tools [1,2]. Considering the destructive effect of the excessive use of fossil fuels on the environment and the health of society with the production of greenhouse gases and also the exhaustibility of these fuel sources, there is a strong need to find new clean fuels and renewable resources [3,4]. Different types of fuel cells, solar cells, electrochemical batteries, and supercapacitors are among the latest energy storage and production tools [5]. The industrialization of these devices and the detailed understanding of how energy is produced and stored require the involvement of different sciences. Indeed, this field is at the frontier of modern research and gathers the attention of many scientists, involved in activities that range from the catalyst and membrane synthesis to the assembling of equipment to be placed in portable devices and cars, etc.

There are different types of fuel cells, some of the most common ones are direct methanol fuel cells (DMFCs), polymer electrolyte membranes (PEMs), alkaline fuel cells (AFCs), phosphoric acid fuel cells (PAFCs), molten carbonate fuel cells (MCFCs), solid oxide fuel cells (SOFCs), and reversible fuel cells, whose main difference is in the operating temperature and the type of electro-chemical reactions, electrolytes, and fuel required and catalysts used in their anode and cathode. Alcohol fuel cells are particularly popular due to their low operating temperature [6], high energy density [7], and small dimensions. Among the fuels used in fuel cells, methanol and ethanol have attracted the attention of scientists due to their cheapness, availability, easy production, and safe storage and transportation [8,9,10]. However, the toxicity of methanol and the relatively low evaporation temperature of both alcohols are among the disadvantages of these fuels [11].

Fuel cells convert chemical energy into electrical energy [12]. In this regard, the oxidation of alcohols (methanol and ethanol) occurs in the anode, and oxygen reduction occurs in the cathode. Between the anode and the cathode, there is a polymer membrane, usually Nafion, which is responsible for proton exchange [13].

So far, valuable efforts have been made to introduce catalysts for use in the anode and cathode of alcohol fuel cells. Several efficient and engineered catalysts have been introduced; however, none of them can compete with catalysts such as platinum, palladium, and ruthenium. Alcohol fuel cells based on these catalysts are expensive and have a gradual loss of electrocatalytic activity in alcohol oxidation [14].

Three categories can be considered in the classification of catalysts for the oxidation process of alcohols. In the first category, there are catalysts such as platinum, palladium, and ruthenium in pure form and with different morphologies. Although they are very efficient, their high price is a big problem for commercialization. In the second category, very small amounts of expensive catalysts are combined or hybridized with cheap and electroactive materials such as conductive polymers, carbons, and metal-organic frameworks (MOFs). In this way, although efficiency decreases, the price of catalysts can be brought down significantly. The third category of attractive and inexpensive catalysts that cannot compete with platinum-based catalysts (and other related families) are platinum-free catalysts. In the synthesis process, it should be noted that the proposed catalyst must have two very important properties: a relatively high electrochemically active surface area and acceptable electrical conductivity. Among these catalysts, we can mention the combination of various materials such as metal oxides and sulfides, different types of carbons, conductive polymers, zeolites, and MOFs [15]. Metal oxides have shown good electrocatalytic activity. Their hybridization and composition with other materials can improve the electrochemically active surface area and electrical conductivity, thus paving the way for new inexpensive catalysts. Carbon, as one of the most abundant elements in nature, is always available, and the synthesis of its derivatives is not a difficult task. From carbon derivatives, we can mention multi-walled carbon nanotubes (MWCNTs), single-walled carbon nanotubes (SWCNTs), hollow carbon spheres (HCNS), biochar carbon, different types of activated carbon, and reduced graphene oxide (RGO). Each of the mentioned types of carbon when added to the catalyst structure improves electrical conductivity, increases the electrochemical active surface area of the catalyst, and generally facilitates the electrochemical processes. Composites consisting of several metal oxides and their hybrids with reduced graphene oxide (rGO) can be a suitable choice for the synthesis of new catalysts. In such catalysts, the synergistic effect of metal oxide and a type of carbon with an excellent active surface area and suitable electrical conductivity can also be exploited [16,17,18].

The review of new scientific studies shows that zirconium oxide (ZrO_2_) [19,20,21,22] and nickel oxide (NiO) [23,24,25] are among the most widely used catalysts based on metal oxides in various fields. The use of these catalysts in the structure of various types of solar cells [26], processes such as water splitting, hydrogen evolution reactions (HERs) [27], oxygen reduction reactions (ORRs) [28], etc., in the field of energy production, as well as the wide application of these materials in the structure of the electrodes of various types of electrochemical batteries, such as lithium, manganese, and zinc-air batteries [29,30], and in the structure electrodes of supercapacitors [31,32] in the field of energy storage, shows the wide capability of these materials in modern electrochemistry. In addition, we must mention the wide application of ZrO_2_ and NiO in other electrochemical applications such as electrochemical biosensors [33,34] and electrochemical detection of various drugs [35,36], antibacterial and photocatalytic properties [37,38,39], as well as in the oxidation process of many materials such as urea, glucose, various sugar alcohols, etc. [40,41]. 

ZrO_2_ and NiO as catalysts in the form of composites with other materials (both expensive catalysts such as platinum and palladium and other inexpensive materials and substrates, including metal oxides and sulfides or conductive polymers) have been studied for the oxidation of alcohols [22,42,43,44,45,46,47,48,49]. However, catalysts including both ZrO_2_ and NiO have not been tested in the oxidation of methanol and ethanol alcohols. For this purpose, we synthesized a nanocatalyst consisting of both zirconium and nickel metal oxides by a hydrothermal method. To simultaneously improve the electrical conductivity and the electrochemical active surface, a hybrid of ZrO_2_/NiO with reduced graphene oxide (rGO) was prepared in the same way and in one step. In this research, in addition to investigating the ability of ZrO_2_/NiO nanocatalysts in the oxidation process of methanol and ethanol alcohols, we investigated the improvement in catalyst performance by adding rGO to the ZrO_2_/NiO structure.

## 2. Materials and Methods

### 2.1. Materials and Equipment

All the materials used in this research, including zirconium nitrate Zr(NO_3_)_4_•5H_2_O and nickel nitrate Co(NO_3_)_2_•6H_2_O, PEG, H_2_O_2_, methanol, and potassium hydroxide (KOH) with purity > 99%, were purchased from Aldrich Company (Wyoming, IL, USA). X-ray diffraction (XRD) analysis was performed with an XRD device, Philips PW1800, and scanning electron microscope (SEM) images were prepared with SEM—TESCAN. Bright-field transmission electron microscopy (BF-TEM) was performed with JEM-1400Plus, with thermionic source (LaB6), operated at 120 kV. Electrochemical analyses were performed with the potentiostat/galvanostat Autolab 302 N (Herisau, Switzerland) with a three-electrode system.

### 2.2. Synthesis of Nanocatalysts

For the synthesis of ZrO_2_/NiO, 0.15 g of zirconium nitrate (Zr(NO_3_)_4_•5H_2_O) and 0.25 g of nickel nitrate (Ni(NO_3_)_2_·6H_2_O) were mixed in 30 mL of deionized water for 20 min with a magnetic stirrer. Then, 0.15 mL of PEG-400 solution and 0.1 mL of H_2_O_2_ were added to the solution, and stirring was continued for another 10 min. The resulting solution was poured into a reactor with a capacity of 50 mL and put in the oven for 14 h at a temperature of 200 °C. The reactor was cooled at room temperature. The product was washed several times with deionized water and ethanol and dried at 80 °C for 8 h and then calcined at 350 °C for 3 h. The resulting powder was ZrO_2_/NiO. For the synthesis of ZrO_2_/NiO/rGO, we followed exactly the same method as for the synthesis of ZrO_2_/NiO, with the difference that in the first, 0.2 g of graphene oxide (GO) was added to the zirconium and nickel precursors.

## 3. Results

### 3.1. Characterization of Nanocatalysts

**Figure 1:**
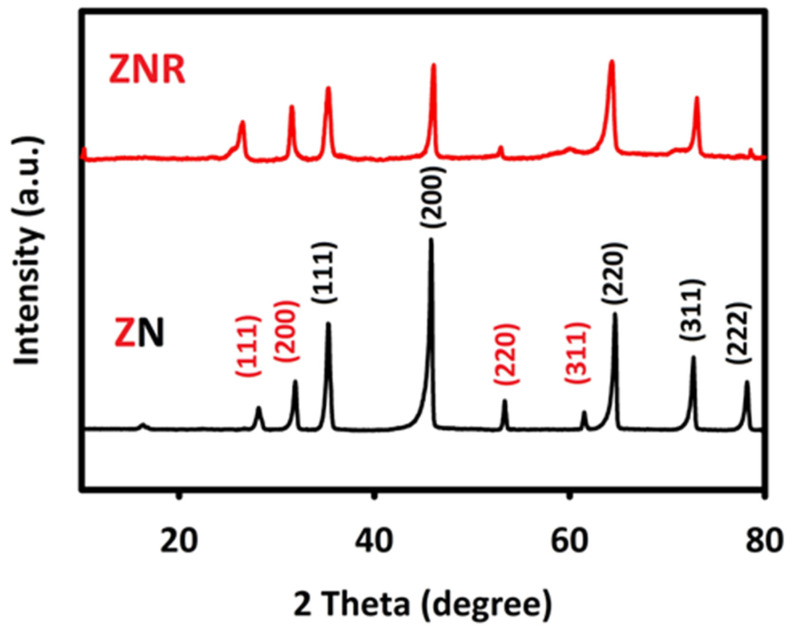
XRD pattern of ZrO_2_/NiO (dark curve) and ZrO_2_/NiO/rGO (red curve).

**Figure 2:**
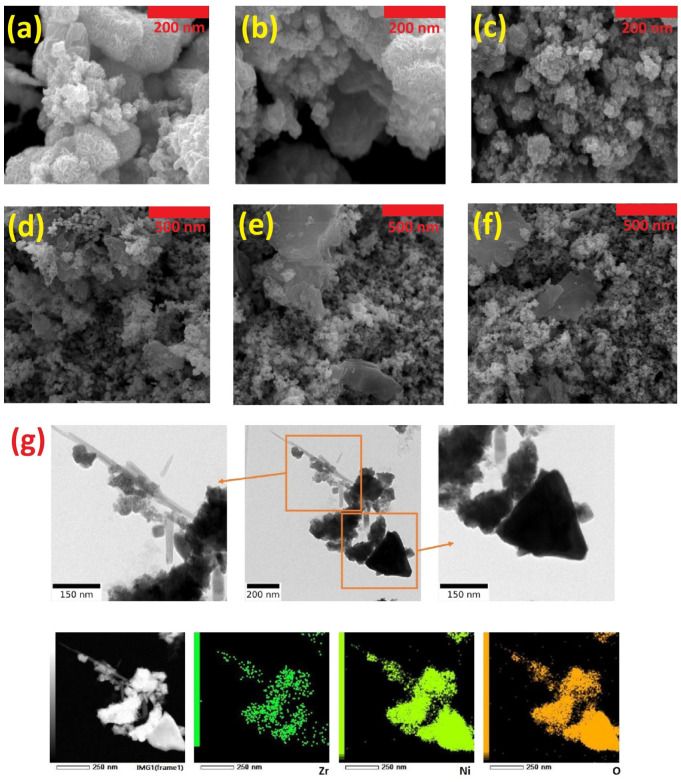
SEM images of ZrO_2_/NiO (**a**–**c**) and ZrO_2_/NiO/rGO (**d**–**f**). BF-TEM images and the element mapping patterns of ZrO_2_/NiO (**g**).

To investigate the crystal structure and surface morphology of the synthesized nanocatalysts, X-ray diffraction (Figure 1) and SEM analyses (Figure 2) were performed, respectively. In the X-ray diffraction pattern of ZrO_2_/NiO (ZN), the characteristic peaks of ZrO_2_ are observed at the diffraction angles of 30, 35.2, 50.2, and 60.3, which correspond to the (111), (200), (220), and (311) crystal planes, which is in complete agreement with (JCPDS, No.49-1642) [48]. The diffraction angles of NiO are also seen at 37.2, 43.2, 62.9, 75.2, and 79.4, which correspond to the (111), (200), (220), (311), and (222) crystal planes with (JCPDS, No. 04-0835) [50]. In the XRD pattern of ZrO_2_/NiO/rGO (ZNR), in addition to observing the diffraction peaks of ZrO_2_ and NiO, we see a relatively wide peak at the diffraction angle around 26 degrees, which belongs to reduced graphene oxide [51].

Scanning electron microscope (SEM) images of ZrO_2_/NiO (ZN) and ZrO_2_/NiO/rGO (ZNR) catalysts were prepared to investigate the surface morphology. Figure 2a–c, b belong to the SEM images of ZrO_2_/NiO on the scale of 200 nm. The relatively porous morphology of this nanocatalyst is clear in these figures. The presence of porosity in the structure of the catalysts causes the creation of channels and shortcuts that facilitate the oxidation process of alcoholic fuels and a faster and deeper contact of the fuel with the core of the catalyst. The SEM images of the ZrO_2_/NiO/rGO catalyst are shown in Figure 2d–f at a scale of 500 nm. The ZrO_2_/NiO nanocatalyst was also analyzed by BF-TEM and mapping related to this analysis. Figure 2g shows the BF-TEM of the catalyst in two scales of 200 and 150 nm. By examining the mapping of ZrO_2_/NiO, which was performed at the scale of 250 nm, the presence of the zirconium, nickel, and oxygen elements in the structure of the catalyst is shown. In addition, the existence of two different structures of ZrO_2_ and NiO in the catalyst can also be found. The darker parts are related to ZrO_2_, and the relatively lighter parts are NiO.

### 3.2. Electrochemical Studies

#### 3.2.1. Electrode Preparation

To perform electrochemical tests, a glassy carbon electrode (GCE) modified with a catalyst was used as a working electrode. A Ag/AgCl electrode and platinum wire with a diameter of 1 mm were used as reference and auxiliary electrodes, respectively. To prepare the working electrode, 0.08 g of catalysts (ZrO_2_/NiO and ZrO_2_/NiO/rGO) was dispersed in 0.5 mL of a solution containing water, Nafion (5%), and isopropyl alcohol by ultrasonication for 30 min. Then, 3 microliters of the obtained uniform slurry was put on the GCE surface.

#### 3.2.2. Investigating the Behavior of ZrO_2_/NiO and ZrO_2_/NiO/rGO Nanocatalysts for MOR and EOR Processes in an Alkaline Environment

**Figure 3:**
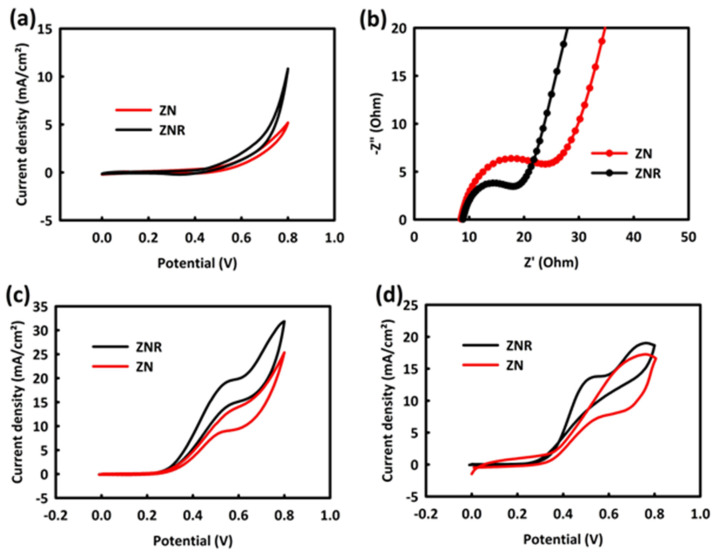
CV curves (**a**) and EIS plots (**b**) of ZrO_2_/NiO and ZrO_2_/NiO/rGO in 0.5 M KOH. CV curves of ZrO_2_/NiO and ZrO_2_/NiO/rGO in 0.5 M KOH/0.1 M methanol (**c**) and 0.5 M KOH/0.1 M ethanol (**d**).

The electrochemical studies were started by performing cyclic voltammetry (CV) and electrochemical impedance spectroscopy (EIS) analyses in an alkaline environment (0.5 M potassium hydroxide (KOH)). The CV analysis of the modified electrode with ZrO_2_/NiO (ZN) and ZrO_2_/NiO/rGO (ZNR) nanocatalysts in the potential range of 0 to 0.8 V with a scan rate of 20 mV/s is shown in Figure 3a. In these graphs, no oxidation peak is seen, and only a faradic current is observed, whose value for ZNR is higher than ZN. Figure 3b belongs to the EIS analysis and the equivalent circuit related to ZN and ZNR nanocatalysts in 0.5 M KOH (without the presence of methanol and ethanol alcohol). According to this analysis, the charge transfer resistance (Rct) for ZN is approximately 19 Ω, and for ZNR, it is about 12 Ω. Both CV and EIS analyses indicate an improvement in the electrocatalytic properties of ZNR nanocatalysts compared to ZN. This superiority in electrocatalytic activity can be related to the effective presence of rGO in the ZNR structure. rGO facilitates electrochemical processes by increasing the electrochemical active surface area and improving electrical conductivity [13,25].

In the next step, the behavior of the catalysts in the presence of 0.1 M methanol was investigated. According to Figure 3c, both nanocatalysts show an oxidation peak, which confirms their ability in the MOR process. Moreover, the ability of nanocatalysts in the EOR process was investigated with the same procedure. The electrochemical behavior of two nanocatalysts in an alkaline environment and in the presence of ethanol (0.5 M KOH/0.1 M ethanol) is presented in Figure 3d. Both nanocatalysts have a relatively good potential in the EOR process as confirmed by the presence of oxidation peaks.

**Figure 4:**
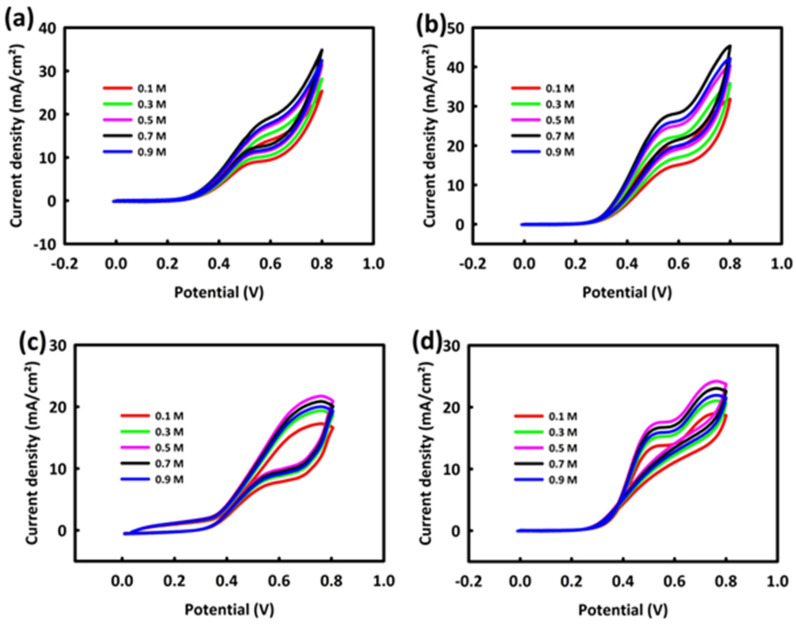
CV from ZrO_2_/NiO (**a**) and ZrO_2_/NiO/rGO (**b**) in 0.5 M KOH/different concentrations of methanol. CV from ZrO_2_/NiO (**c**) and ZrO_2_/NiO/rGO (**d**) in 0.5 M KOH/different concentrations of ethanol.

In the following, the concentration of alcohols in the MOR and EOR processes was optimized by the electrode modified with two ZN and ZNR nanocatalysts. For this purpose, a solution containing 0.5 M KOH and different concentrations of methanol (0.1, 0.3, 0.5, 0.7, and 0.9 M) was prepared. Figure 4a shows the behavior of a ZN nanocatalyst in different concentrations of methanol in CV analysis at a scan rate of 20 mV/s. As can be seen, with the increase in the concentration of methanol up to 0.7 M, the oxidation peak has an upward trend, and at the concentration of 0.9 M methanol, the current density of the oxidation peak decreases; exactly the same trend is observed for ZNR (Figure 4b).

The behavior of both nanocatalysts was investigated in the alkaline environment of 0.5 M KOH and in different concentrations of ethanol (0.1, 0.3, 0.5, 0.7, and 0.9 M) at the scan rate of 20 mV/s. Figure 4c shows the behavior of ZN in different concentrations of ethanol. As can be seen, the oxidation current density has an upward behavior up to the concentration of 0.5 M ethanol, and from this concentration onward, a decrease in the current density is seen. A similar behavior is observed for ZNR in different concentrations of ethanol (Figure 4d).

In general, the behavior of both nanocatalysts in different concentrations of methanol and ethanol indicates that with the increase in the alcohol concentration up to a critical concentration, the peak oxidation current density value increases, and after this concentration, the catalyst surface seems to be saturated by the by-products of the ethanol and methanol oxidation, and as a result, alcohol fuel cannot adsorb well on the surface of the catalyst and penetrate into the core of the catalyst. By-products of the oxidation of alcohols by saturating the surface of the catalyst prevent the easy exchange of electrons and, as a result, avoid the generation of an exchange current between the modified electrode and the alkaline solution containing alcoholic fuels. 

**Figure 5:**
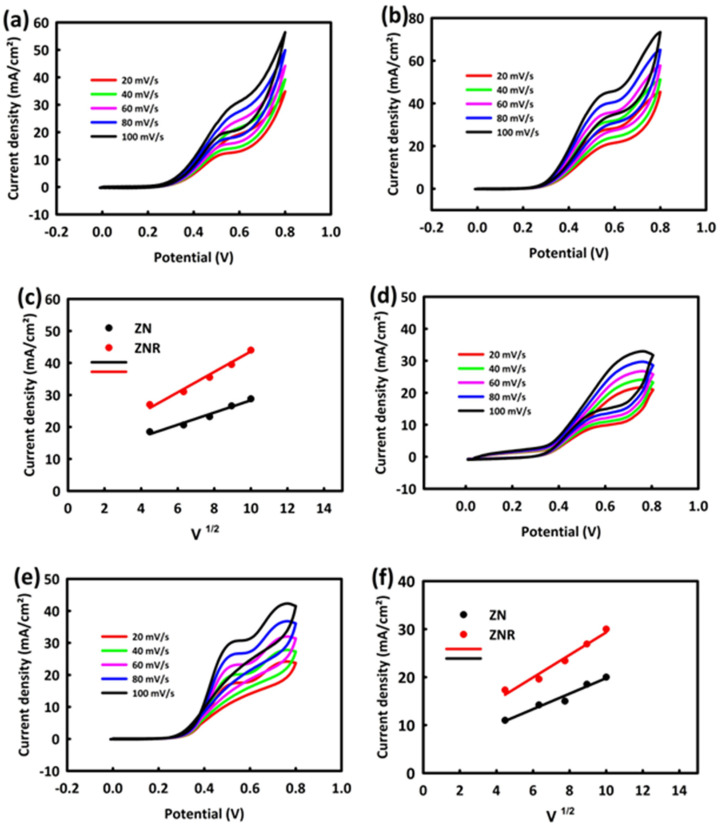
CV from ZrO_2_/NiO (**a**) and ZrO_2_/NiO/rGO (**b**) in 0.5 M KOH/0.7 M methanol/different scan rates. CV from ZrO_2_/NiO (**d**) and ZrO_2_/NiO/rGO (**e**) in 0.5 M KOH/0.5 M ethanol/different scan rates. The plot of the square root of scan rate in terms of maximum current density for ZrO_2_/NiO and ZrO_2_/NiO/rGO in MOR (**c**) and in EOR (**f**).

After optimizing the concentration of alcohols, the behavior of nanocatalysts in the optimal concentration of methanol and ethanol and at different scan rates was investigated. Figure 5a shows the behavior of ZN in 0.5 M KOH/0.7 M methanol solution at different scan rates (20, 40, 60, 80, and 100 mV/s). As can be seen, with the increase in the scan rate, the oxidation peak current density indicates an upward trend, and the same behavior is seen for ZNR nanocatalysts at different scan rates (Figure 5b). The maximum current density as a function of the square root of the scan rate (Figure 5c) for two nanocatalysts is fitted with a straight line with R^2^ = 0.998 and R^2^ = 0.996 for ZN and ZNR, indicating the diffusion-control mechanism in the MOR process.

The mechanism of the MOR by nanocatalysts can be proposed as a six-electron mechanism as follows [52]:

$$Catalyst+CH_{3}OH\to Catalyst-CH_{3}OH_{ads}$$

$$Catalyst-CH_{3}OH_{ads}+4OH¯\to Catalyst-{\left(CO\right)}_{ads}+4H_{2}O+4e¯$$

$$Catalyst+OH¯\to Catalyst-OH_{ads}+e¯$$

$$Catalyst-CO_{ads}+Catalyst-OH_{ads\;}+OH¯\;\to Catalyst+CO_{2}+H_{2}O+e¯$$

Similarly, the behavior of ZN and ZNR nanocatalysts in a 0.5 M KOH/0.5 M ethanol solution and at different scan rates was evaluated. Here too, the peak oxidation current density of both nanocatalysts increases with an increasing scan rate in the range of 20 to 100 mV/s (Figure 5d,e). The straight-line fit (with R^2^ = 0.996 and R^2^ = 0.994 for ZN and ZNR, respectively) of the maximum current density versus the square of the scan rate in the EOR process shows the diffusion-control mechanism (Figure 5f).

The proposed mechanism of ethanol oxidation by nanocatalysts can be suggested as follows [53]:

$$CH_{3}CH_{2}OH+16OH^{-}\;\to \;2CO_{3}^{2-}+11H_{2}O+12e^{-}\;$$

$$CH_{3}CH_{2}OH+5OH^{-}\;\to \;CH_{3}COO^{-}+4H_{2}O+4e^{-}$$

**Figure 6:**
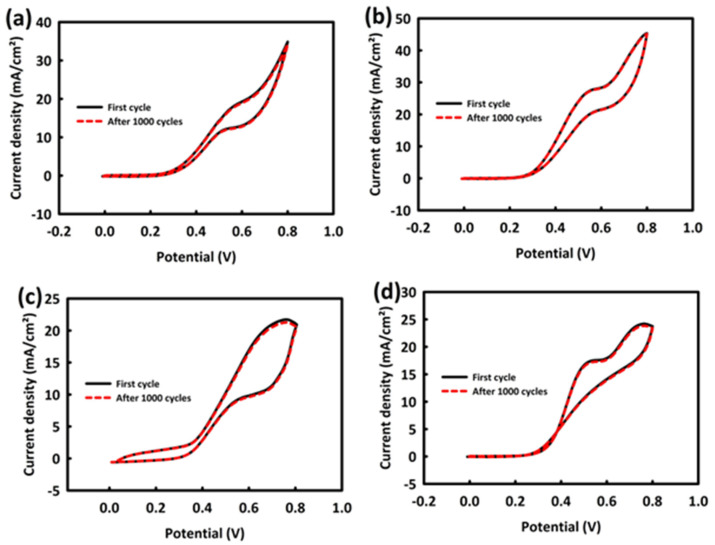
Cyclic stability for ZrO_2_/NiO (**a**) and ZrO_2_/NiO/rGO (**b**) in MOR and cyclic stability for ZrO_2_/NiO (**c**) and ZrO_2_/NiO/rGO (**d**) in EOR.

The stability of ZN and ZNR nanocatalysts in the MOR and EOR processes was determined by performing 1000 consecutive CV cycles in a 0.5 M KOH/optimal concentration of alcohols and at a scan rate of 20 mV/s. Figure 6a,b are related to the stability of ZN and ZNR in the MOR process. ZN has 98.3% stability after 1000 consecutive cycles, and ZNR shows 99.5% stability, which are very good values.

Figure 6c,d show the stability of ZN and ZNR nanocatalysts in the EOR process after 1000 consecutive CV cycles. Cyclic stability after this number of cycles is 97.9% for ZN and 98.5% for ZNR.

By investigating the cyclic stability of nanocatalysts in the MOR and EOR processes, a drop in current density is observed in the initial cycles, but, after that, with the complete penetration of methanol and ethanol into the core and structure of the catalyst and full contact between them, very good stability in current density is achieved. The comparison of the stability of nanocatalysts in the MOR and EOR processes shows the relative superiority of ZNR nanocatalysts compared to ZN in terms of cyclic stability. This superiority can be related to the presence of RGO in the catalyst structure. By increasing the electrochemically active surface, rGO facilitates the oxidation process of alcohols, and by making its active surface available to the fuel, it causes faster cyclic stability of the nanocatalyst in the oxidation process.

**Figure 7:**
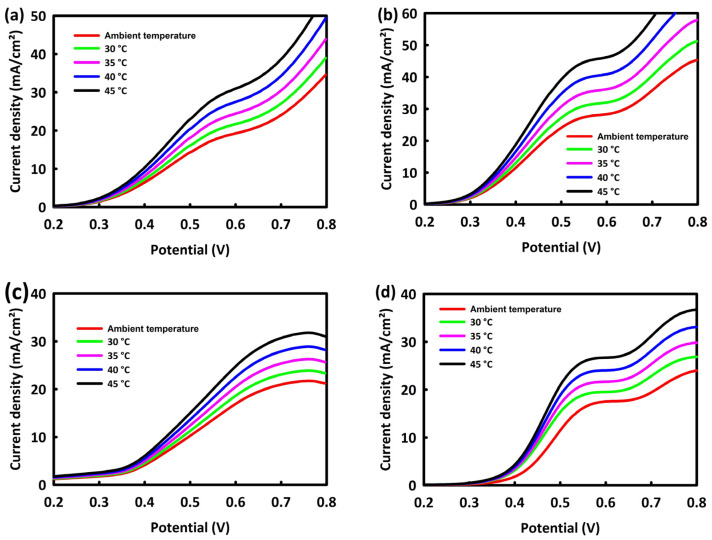
LSV curves of ZrO_2_/NiO (**a**) and ZrO_2_/NiO/rGO (**b**) in MOR process and LSV of ZrO_2_/NiO (**c**) and ZrO_2_/NiO/rGO (**d**) in EOR process at different temperatures.

The behavior of catalysts in the process of methanol and ethanol oxidation at different temperatures was investigated by performing linear sweep voltammetry (LSV) analysis at a scan rate of 20 mV/s and in the temperature range of ambient temperature up to 45 °C. An increase in temperature improves the oxidation process of alcohols, and according to the linear relationship between temperature and current density, an increase in the peak oxidation current density is observed with increasing temperature. Figure 7a,b show the behavior of ZN and ZNR nanocatalysts at different temperatures. The increase in temperature facilitates the kinetics of the MOR and, as a result, increases current density. In the process of ethanol oxidation, the same behavior is observed for ZN and ZNR nanocatalysts at different temperatures (Figure 7c,d). The linear relationship between temperature and current density can be observed in the LSV diagrams. Hence, an increase in the oxidation current density occurs in both methanol and ethanol oxidation processes with the rising temperature.

In Table 1, the efficiency of the ZrO_2_/NiO/rGO nanocatalyst in the methanol and ethanol oxidation process is compared with other recent articles. The oxidation peak potential and current density of ZrO_2_/NiO/rGO are comparable with other similar works.

## 4. Conclusions

Due to their high electrocatalytic activities, zirconium oxide and nickel oxide were combined with reduced graphene oxide to form novel catalysts. Adding rGO to the catalyst structure facilitates the oxidation process of alcohols. We investigated the behavior of ZrO_2_/NiO and ZrO_2_/NiO/rGO in the MOR and EOR processes. We demonstrated that ZrO_2_/NiO/rGO is an attractive, cheap, and stable nanocatalyst with a relatively easy synthesis. It is worth mentioning that although these new nanocatalysts cannot compete yet with catalysts based on noble and expensive metals, such as platinum, ruthenium, and palladium, they can offer a good option for alcohol oxidation in fuel cells. The methanol and ethanol current densities obtained for ZrO_2_/NiO/rGO were 26.6 mA/cm^2^ and 17.3 mA/cm^2^, respectively. The interesting point is that the potential peak for both processes is seen at approximately 0.52 V, and the proposed catalyst has very good stability in both MOR and EOR processes. Considering that ZrO_2_ and NiO have always been prominent in the field of catalysts and have shown very good electrocatalytic activity, our research team aims to investigate the capability of these catalysts in different fields of energy and electrochemical determination of some drugs and alcohol in our future studies. The preliminary results of the performed electrochemical analyses show the excellent potential of this catalyst in the process of oxidation and sensing of sorbitol, urea, glycerol, and glucose. Moreover, the ability of this material as a supercapacitor electrode and electrochemical battery can also be evaluated.

## Figures and Tables

**Table 1:** Comparison of MOR and EOR performances of ZrO_2_/NiO/rGO nanocatalyst with other research.

Electrocatalyst	Electrolyte Composition	Peak Potential (V)	Current Density (mA cm^–2^)	Scan Rate (mV/s)	Reference
ZrO_2_/NiO/rGO	0.7 M Methanol/0.5 M KOH	0.52	26.6	20	This work
ZrO_2_/NiO/rGO	0.5 M Ethanol/0.5 M KOH	0.52	17.3	20	This work
ZnFe_2_O_4_-ZrO_2_/Pt	1 M Methanol/0.5 M KOH	0.35	104.75	50	[54]
Zr-MOF@PANI/Ni-NPs/GCE	0.5 M Methanol/1 M NaOH	0.75	291.6	100	[55]
Ni@Au@Pd/rGO	1 M Ethanol/2 M KOH	0.6	8.85	50	[56]
NiO-Y_2_O_3_/FTO	0.6 M Methanol/0.5 M NaOH	0.65	6.2	100	[57]
NiO-ZrO_2_/FTO	0.6 M Methanol/0.5 M NaOH	0.65	10	100	[57]
MOF-74(Ni)/NiOOH	1 M Methanol/1 M KOH	0.6	27.62	50	[58]
Mn_3_O_4_-CeO_2_-rGO	0.8 M Methanol/1 M KOH	0.51	17.7	90	[5]
Mn_3_O_4_-Co_3_O_4_-rGO	1 M Methanol/1 M KOH	0.48	16.5	100	[59]
Mn_3_O_4_-Co_3_O_4_-rGO	0.7 M Eethanol/1 M KOH	0.55	5.7	60	[59]
MnCo_2_O_4_/NiCo_2_O_4_/rGO	2 M Methanol/2 M KOH	0.58	24.76	20	[60]
Au-NiOx/g-C3N4	0.5 M Methanol/1 M KOH	0.35	32.5	Not Reported	[61]
MnO_2_–NiO–MWCNTs	0.5 M EtOH/1 M KOH	0.55	148 μA/cm2	50	[62]
Pd–Ni–Fe/MnO_2_/Vulcan	1 M Ethanol/0.2 M KOH	+0.05–+0.3	3.03	100	[63]

## Data Availability

The data presented in this study are available on request from the corresponding authors.
